# Colour thresholds in a coral reef fish

**DOI:** 10.1098/rsos.160399

**Published:** 2016-09-21

**Authors:** C. M. Champ, M. Vorobyev, N. J. Marshall

**Affiliations:** 1 Queensland Brain Institute, The University of Queensland, Brisbane, Australia; 2 Department of Optometry and visual science, Auckland University, Auckland, New Zealand

**Keywords:** coral reefs, colour vision, visual thresholds

## Abstract

Coral reef fishes are among the most colourful animals in the world. Given the diversity of lifestyles and habitats on the reef, it is probable that in many instances coloration is a compromise between crypsis and communication. However, human observation of this coloration is biased by our primate visual system. Most animals have visual systems that are ‘tuned’ differently to humans; optimized for different parts of the visible spectrum. To understand reef fish colours, we need to reconstruct the appearance of colourful patterns and backgrounds as they are seen through the eyes of fish. Here, the coral reef associated triggerfish, *Rhinecanthus aculeatus*, was tested behaviourally to determine the limits of its colour vision. This is the first demonstration of behavioural colour discrimination thresholds in a coral reef species and is a critical step in our understanding of communication and speciation in this vibrant colourful habitat. Fish were trained to discriminate between a reward colour stimulus and series of non-reward colour stimuli and the discrimination thresholds were found to correspond well with predictions based on the receptor noise limited visual model and anatomy of the eye. Colour discrimination abilities of both reef fish and a variety of animals can therefore now be predicted using the parameters described here.

## 1. Background

The fish that inhabit the coral reefs of the world live in an environment where light is abundant and a stunning assemblage of colours and patterns are present. This diversity of colour on the reef has long been noted [1–3], and in more recent decades investigated via methods such as spectroscopy, anatomical investigation and more recently behavioural testing [4–7]. Explanations for coloration include either camouflage or display, with some fish species combining both for various tasks in predation, survival and mate choice [8]. However, understanding the reasons behind a given species choice of colour pattern is complicated by a lack of understanding of what reef fish ‘see’ when they look at the reef. Given the differences in human and fish photoreceptors and spectral sensitivities [9], it is almost certainly not the same as what humans perceive. An important step forward then in understanding the colourful patterns of reef fish is to model their colour discrimination thresholds. Here, we show that the behavioural thresholds of a reef fish can be modelled using data derived from the anatomy of the eye and the absorbance of its photoreceptors. Because such data are available for many species, the method employed here can be used to model colour discrimination in many animals.

Much of what we know about fish colour vision is based on studies of freshwater fish such as the zebrafish (*Danio rerio*) and the goldfish (*Carassius auratus*) [10]. These studies have revealed that colour discrimination in these species displays general features such as as colour constancy [11] and chromatic adaptation [12], inhibitory interactions between receptor mechanisms, indicating colour opponent processing [13] and colour blindness of motion detection [14]. The colour vision of marine fish and, in particular, coral reef fish has been investigated in much less detail. Only recently, it has been demonstrated that coral reef fish are capable of using colour to discriminate light stimuli [6,15]. The vision of the *Rhinecanthus aculeatus*, a type of marine triggerfish, has been studied in some detail using both behavioural and anatomic methods [16–18]. *Rhinecanthus aculeatus*' eyes have the two distinct types of cones: the single cones and the double cones, which house three types of visual pigment [19]. A behavioural study of *R. aculeatus* colour discrimination was conducted to show that the signals of the two members of double cones are compared along with the single cone sensitivity to provide a trichromatic visual system [16]. Further to this, it is also known that spatial acuity in *R. aculeatus* is significantly worse than the limit predicted from the spacing of cones in its retinal array [18]. This may indicate that summation of signals of individual cones leads to improvement of the signal-to-noise ratios of receptor mechanisms [18].

Colour discrimination can be modelled using the receptor noise limited (RNL) model that assumes colour is processed by colour opponent mechanisms [20]. An important parameter of the RNL model is the level of noise in photoreceptor mechanisms, which can be estimated by measuring the total numbers of photoreceptors, and their physical dimensions [20,21]. Because data for estimates of the levels of noise of receptor mechanisms are available for many animals, the RNL model is used in ecological studies (e.g. [20,22–24]). The RNL model predicts the shape of spectral sensitivity and/or wavelength discrimination curves in many animals, including humans, birds and insects [20]. However, the validity of the RNL model has not been tested for marine fish. To determine the magnitude of threshold distance, the RNL model needs to be calibrated against behavioural thresholds [20,21]. So far, the RNL model has been calibrated against colour thresholds for human beings, some species of birds [25,26] honeybees [21] and a butterfly [27].

The aims of our study were firstly to test if the RNL model describes colour thresholds in a fish and then to calibrate the model against behavioural data for a marine triggerfish *R. aculeatus*. We tested the validity of the model by comparing the discriminability of colours in two different directions in the colour plane. We calibrate the model by determining the magnitude of colour distance corresponding to behavioural threshold. The discriminability predicted from eye size and photoreceptor dimensions agrees with the results of behavioural test, and we conclude that the model describes colour discrimination in this species well.

## 2. Material and methods

### 2.1. Animals

**Figure 1.**
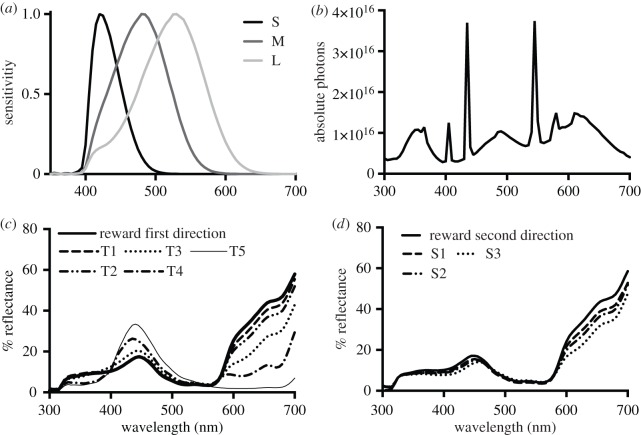
(*a*) The spectral sensitivities of the *R. aculeatus* (from Cheney *et al.* [19]). (*b*) The illumination of the test room in absolute photons. (*c*) The reflectance spectra of the colours in the first series of test colours (T). (*d*) the reflectance spectra of the colours in the second series of test colours (S).

We used the reef species of triggerfish, *R. aculeatus*. Fish were between 8 and 12 cm in size and collected around Lizard Island, northern Queensland, Australia. Fish were caught with permission from the Great Barrier Reef Marine Park Authority (permit number G03/9374.1) and the Queensland Fisheries Service (permit number PRM01599G). Individual fish were kept in separate tanks and water temperature kept constant (23–26°C). The experimental room was illuminated by standard 60 W florescent tubes and by 60 W Samsung blacklights (figure 1). Fish were fed with commercial fish flake (Flake Frenzy, HBH enterprises, Springville, USA) prior to training.

### 2.2. Stimuli

Two sets of stimuli spanning two different directions in the colours plane were used. The stimuli S were arranged so that they differed from the reward stimulus predominantly in the direction corresponding to the difference signal of long wavelength and medium wavelength cones. The stimuli T were arranged so that they differed predominantly in the direction corresponding to the variation of short wavelength cone signal. Colours used in the experiment were created on a standard computer monitor by altering the RGB values for each colour. To produce the stimuli, these colours were printed as 5 cm circles on photographic paper using an Epson 1290 inkjet printer, and then laminated [28]. The reflectance spectra of laminated stimuli and the illumination spectrum were measured using an Ocean Optics USB4000 spectrometer and a 150 W xenon arc lamp (Thermo Oriel model: 66906).

### 2.3. Behavioural experiment

**Figure 2.**
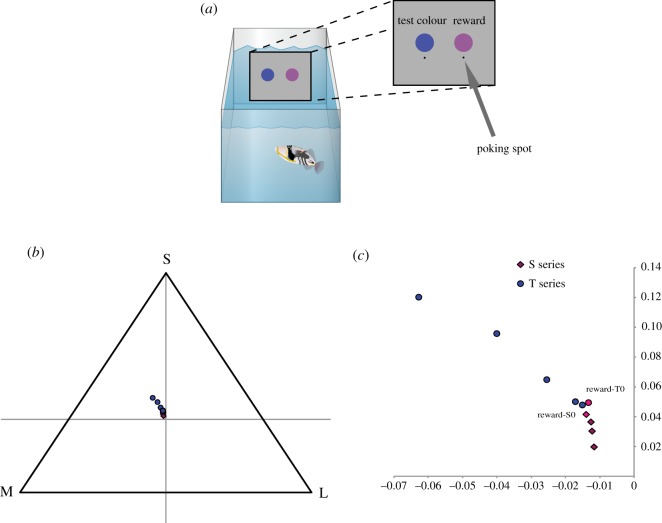
Test stimuli and experimental set-up. Three fish were trained to discriminate the reward colour from the other test colours. Test stimuli were designed so that they became progressively more similar to the reward. Two sets of colours were designed to test the fish in different parts of its perceptual colour space, the T series and the S series. (*a*) Test set-up, fish had to poke at the ‘poking’ spot below the stimulus to make a choice for a given stimulus. (*b*) Test colours in a Maxwell triangle showing their relative positions in colour space. (*c*) Close-up of colours in Maxwell triangle.

Behavioural experiments were carried out under the University of Queensland Ethics and AEC approval number: SBS/738/08/ARC. Test fish had to discriminate between two presented colours. Fish were trained to one ‘reward’ colour and tested against an alternative second colour. Test colours were designed so that they would get progressively more similar, chromatically, to the reward colour*.* A test stimulus which fish were unable to discriminate from the reward was indicative of a ‘perceptual distance’ at which the animal could no longer discriminate two colours. A grey plastic board (24 × 20 cm, 5 mm thick) was placed into the tank. Stimuli circles were fixed to this board in set positions and orientated so that they were evenly spaced and level on the board. One centimetre below each stimulus was a ‘poking spot’ 0.3 cm in diameter, which the fish poked to make a choice for a particular stimulus (figure 2*a*).

### 2.4. Training and testing

The method used is that of Pignatelli *et al.* [16], which was modified from that of Siebeck *et al.* [29]. Training was identical to testing. The two stimuli, the ‘rewarded’ and the alternative were presented to the fish side by side in the front of the aquarium on the grey plastic board (figure 2*a*). To avoid the influence of cues not related to the stimuli, the food reward (a paste made with fish flakes and seawater) was given at the rear end of the aquarium after the fish poked beneath the ‘rewarded’ stimulus. We started to record choices after fish learned to peck at the stimuli at the front of the tank and collect the reward at the rear end of the aquarium. Between tests the stimuli were removed from the aquaria. The position of the stimuli was changed in a random order, but the stimuli never appeared on the same side more than three times in a row [30]. No more than 30 choices a day were conducted for each fish, to prevent loss of motivation and interest in the task.

### 2.5. Analysis of behavioural data

Results were obtained from three individual fish. The dependence of the percentage of correct choices on colour distance, calculated via the RNL model, was fitted using the maximum-likelihood method. Confidence intervals and significance of colour discrimination were calculated using binomial distribution, significance of difference between choice proportions was estimated using Fisher exact test.

### 2.6. Anatomical measurements

Animals were euthanized with an overdose of clove oil in accordance with of the Australian code of practice for the care and use of animals for scientific purposes 2004 and with University of Queensland ethics guidelines (AEC approval number: SBS/738/08/ARC). Pupil and lens size was recorded from 10 individual fish immediately after death using electronic callipers under a microscope. For photoreceptor measurements, seven retinas from four individuals were examined to measure photoreceptor dimensions. Retinas were wholemounted following the methods of Stone [31] and Litherland & Collin [32]. Examination with a Zeiss Axioplan II compound microscope fitted with an *x*-*y*-*z* motorized stage (BioPrecision, LUDL Electronic Products Inc., NY, USA) allowed for estimation of the ratio of double cones to single cones and also measurements of the cross-sectional area of inner segments. The outer segment length of the cones, which is not visible on wholemounts of the retina, was measured using confocal microscopy. To obtain the length measurements, cones were stained following the methods of Pignatelli & Strettoi [33]. Outer segments observed with an Olympus BX61 upright confocal microscope equipped with Olympus UPlanSApo lenses (10×, 20×, 40×), and filters to visualize DiO (3, 3′-dihexadecyloxacarbocyanine-perchlorate; Invitrogen-Life Technologies) and DAPI dilactate (4′, 6-diamidino-2-phenylindole; Invitrogen-Life Technologies) fluorescence.

### 2.7. Modelling colour discrimination

#### 2.7.1. Colour distance and noise of receptors mechanisms

To estimate colour thresholds, we use the RNL model [21]. This model assumes that colour discrimination is achieved by chromatic mechanisms alone (and not brightness) and that the noise originating in cones sets the thresholds. The discriminability of colours *a* and *b* is described by the perceptual distance between colours, Δ*S^*ab*^*, which is calculated as follows:

2.1 $$\Delta S^{ab}=\sqrt{\frac{{\left(\omega_{S}^{ab}(\Delta f_{L}^{ab}-\Delta f_{M}^{ab})\right)}^{2}+{\left(\omega_{M}^{ab}(\Delta f_{L}^{ab}-\Delta f_{S}^{ab})\right)}^{2}+{\left(\omega_{L}^{ab}(\Delta f_{S}^{ab}-\Delta f_{M}^{ab})\right)}^{2}}{{\left(\omega_{S}^{ab}\omega_{M}^{ab}\right)}^{2}+{\left(\omega_{S}^{ab}\omega_{L}^{ab}\right)}^{2}+{\left(\omega_{L}^{ab}\omega_{M}^{ab}\right)}^{2}}}$$

where $\Delta f_{i}^{ab}$ is the difference between the signals *a* and *b* corresponding to receptor mechanism *i* (*i *= S, M, L) and the ‘noise’, $\omega_{i}^{ab}$, is the standard deviation of signals of receptor mechanism *i* when comparing the stimuli *a* and *b*. For a trichromatic system such as that of the triggerfish, S is the short wavelength cone mechanism, M is the medium wavelength cone mechanism and L is the long wavelength cone mechanism. The signal of receptor mechanisms *i* for a stimulus *a*, $f_{i}^{a}$, is a function of the absolute quantum catch, $Q_{i}^{a}$ (the number quanta absorbed per receptive field and integration time by a receptor of a given spectral type) [34].

The RNL model assumes that photoreceptor noise sets discrimination thresholds. Assuming that spatial pooling improves the signal-to-noise ratio for a receptor channel the Weber fraction for a receptor channel can be calculated as follows:

2.2 $$\omega_{i}=\frac{\sigma_{i}}{\sqrt{\eta_{i}}},$$

where *ω* is the Weber fraction, *σ* is the standard deviation of the noise in a single cone channel and *η* is the relative proportion of cone type *i*. The relative proportions of each cone type were derived from the anatomy and were found to be 1 : 2 : 2 for the S, M and L types, respectively. We assumed a standard deviation of noise to be 0.05 which is a reasonable estimate for most animal studies [20].

#### 2.7.2. Calculation of quantum catches

Quantum catches for each stimulus were calculated as follows:

2.3 $$Q_{i}^{a}=\int R_{i}(\lambda )S^{a}(\lambda )I(\lambda )\;d\lambda$$

where *I*(*λ*) is the illumination spectrum, *S^*a*^*(*λ*) is the reflectance spectrum of a stimulus *a, R_i_*(*λ*) is the absolute spectral sensitivity of a receptor channel *i.* Maximum wavelength of absorption for every cone had been measured previously by Cheney *et al.* [19].

To calculate the absolute spectral sensitivity, we follow the method explained by Land [35]:

2.4 $$R_{i}(\lambda )=\nu \tau {\left(\frac{\pi }{4}\right)}^{2}\Delta \rho^{2}D^{2}O(\lambda )A_{i}[1-\exp(-\mu_{i}(\lambda )l],$$

where *O*(*λ*) and *A_*i*_* are the transmittances of the ocular media, *μ_*i*_*(*λ*) is the optical density of the visual pigment type *i*, *l* is the length of the outer segment, *v* is the number of cones per receptive field, *τ* is the summation time, Δ*ρ* is the acceptance angle of a cone and *D* is the pupil diameter. Ocular media measurements for this species were taken from Cheney *et al.* [19]. The optical density of cones was estimated as 0.015 µm following estimates used by Vorobyev [36] and the acceptance angle of the cone was estimated as Δ*ρ* = *d*/*f*, where *d* is the diameter of the receptor and *f* is distance to the nodal point (2.5 mm, calculated from the diameter of the lens via Matthiessen's rule). Integration time was estimated as 40 ms, a reasonable estimate for a marine fish living in bright conditions (flicker fusion frequency of 25 Hz) [37,38].

## 3. Results

In line with previous attempts to train fish with colour tasks [39], *R. aculeatus* proved difficult to train to perform psychophysical tests. From an initial group of seven fish, three fish were trained to discriminate a reward colour stimulus from a series of test stimuli over a four month period (figure 2*a*). These test stimuli were designed so that they became progressively more similar to the reward in terms of colour until they were indistinguishable (figure 2*b*,*c*). The two sets were also designed to test different parts of the animals ‘perceptual colour space’ in such a way that they would approach the same point from different directions in the colour space (figure 2*b*,*c*). Such comparisons can be ‘mapped back’ to the animal's colour world exposing colour contrasts related to fish behavioural choices. For the test, stimuli luminance level was also controlled to ensure the animals could not use ‘brightness’ as a cue for discrimination. The fish were first tested with a series of colours labelled T5, T4, T3, T2 and T1 to identify how chromatically different they were from the reward (T0); T5 being the most different T1 and the most similar to the reward stimulus. All three fish were able to discriminate the colours T5–T2 from the reward with choice frequencies of 86% (*p* < 0.0001, *n* = 270), 87% (*p* < 0.0001, *n* = 270), 86% (*p* < 0.0001, *n* = 270) and 65% (*p* < .0001, *n* = 270), respectively (binomial test). Fish failed to discriminate the reward colour from the T1 test colour with a choice frequency of 50.7% (*p* = 0.42, *n* = 270 binomial).

The fish were then tested with a second series of different colours labelled S3, S2 and S1; S3 being the most different to the reward (S0) and S1 the most similar. In the second direction fish discriminated the colour S3 with a choice frequency of 85.5% (*p* < 0.0001, *n* = 270 binomial). For colours S2 and S1, the choice frequencies were 56.2% (*p* = 0.02, *n* = 270) and 53.7% (*p* = 0.12, *n* = 270), respectively (binomial test).

**Figure 3.**
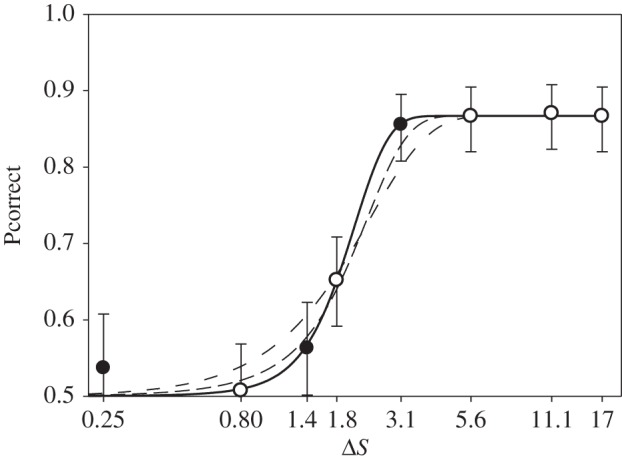
Proportion of correct choices, Pcorrect, as function of colour distance, Δ*S*. The data for all fish are pooled. Filled circles correspond to direction S, open circles correspond to direction T. Error bars indicate 95% CIs (binomial test). The colour distance is plotted on a logarithmic scale. Solid line is the theoretical psychometric function that gives the best fit for the ideal observer model (*t* = 2.85), as described in Vorobyev *et al.* [21]. The thin lines show the psychometric functions for *t* = 1 and 2—where *t* is the response criterion which must be exceeded to detect a stimulus and the threshold of discrimination is taken as 75% correct choices.

To find out if the RNL model [20,22] described colour discrimination, we compared the colour choices determined for the two directions in the colour space with the corresponding colour distances. Colour distances were calculated with the RNL model, here with the addition of the use of photoreceptor and optical anatomy of the eye to fine-tune the estimate of receptor noise. We incorporated the receptor and pupil dimensions into the calculations of perceptual distance. This has rarely been attempted in the past but is a relatively easy addition to the process of describing discrimination ability. Adding these dimensions allowed a better estimate of the noise, and therefore the discrimination threshold between two different colours. Without using this step, the rankings of the choice proportions did not correspond with the calculated distances. This was particularly problematic close to the threshold where without this step the calculated distances for T2 = S2. However, this did not match with the choice proportions observed, as T2 was discriminated significantly better by the fish than S2. After including full eye anatomy, not just photoreceptor number, the ranking of the distances between the rewarded stimuli and the test stimuli (T5 > T4 > T3 > S3 > T2 > S2 > T1 > S1) agree with ranking of the choice proportions (P_T5_ = P_T4_ = P_T3_ = P_S3_ > P_T2_ > P_S2_ > P_S1_ > P_T1_) with one exception—the choice proportion for S1 is higher than for T1. However, the S1 and T1 choice proportions do not differ significantly from each other or from the chance level (figure 3). Therefore, our results indicate that colour discrimination in the direction S and in the direction T can be described by colour distance estimated from anatomy.

## 4. Discussion

Behavioural discrimination limits or thresholds are difficult to obtain due to the time required to train animals to perform behavioural tasks. As previously mentioned, despite long-standing interest in their coloration, only recently has it been behaviourally demonstrated that coral reef fish are capable of using colour to discriminate stimuli [6,8]. In the absence of quantitative data on behavioural discrimination, it is convenient and useful to use the RNL model to assess conspicuousness and colour use. Many ecological studies on birds [23,40,41] and reptiles [42,43] have made such use of the model. In fish too, conspicuousness of potential prey items to fish has been examined a number of times with the model alone [44–46]. However, before this study the validity of the RNL model and its implementation using ocular anatomy had not been verified for marine fish.

The RNL model estimates the perceptual distance between colours in colour space. When colour distance exceeds a certain threshold or ‘just noticeable distance’ the model predicts that colours can be discriminated. Estimates of colour distances can be made in the absence of detailed knowledge about photoreceptor interaction or central colour processing [20,21]. Instead, photoreceptor spectral sensitivity, determined directly through microspectrophotometry, physiology or even estimated using visual pigment genetic sequence data, are used to model colour vision capability [17–19]. However, the RNL model predicts the relation of discriminability of colours to these parameters rather than the absolute threshold. To determine the magnitude of threshold distance, the RNL model needs to be calibrated against behavioural thresholds, as has been achieved here for the first time by determining the magnitude of colour distance corresponding to behavioural threshold. Interestingly, a match between behaviour and combined anatomical/physiological estimates was achieved with the inclusion of photoreceptor dimensions, allowing the relative sensitivity of photoreceptors to be added and indicating that colour discrimination is limited by fluctuations of absorbed photons. For *R. aculeatus* then, as the relation of discriminability predicted photoreceptor dimensions and spectral sensitivities agree well with the results of behavioural tests, we can conclude that the model alone describes colour discrimination sufficiently. Our comparison of behavioural results to anatomy is significant for further assessments of reef fish and other animal colour vision, as it bolsters our confidence in the predictive power of the RNL model with this new addition of simply attained ocular anatomy. While the ‘acid-test’ for any colour vision system is direct behavioural evidence, the methods described here indicate we can at least begin to get closer to a good understanding of colour vision in a range of species where only data on retinal anatomy are available and photoreceptor numbers have been counted.

When considering the behavioural response of the fish it is also of interest that response to stimuli above threshold saturates (for review, see Geisler [47]). Thus, behavioural data could be fitted with psychometric curves via the ideal observer theory [21]. This theory predicts that the dependence of the number of correct choices on colour distance has a sigmoid shape with a steep transition. Stimuli providing signal above the response criterion are discriminated and the stimuli below response criterion are not discriminated. Practically speaking, large chromatic distances do not necessarily equate with a greater ability to discriminate colours or colours that are verifiably more conspicuous. Rather, colours that are sufficiently above threshold appear to be discriminated with the same proficiency by the fish. It should be noted that, in the aquatic environment, water does act as an attenuating medium and gradually makes colours more achromatic over distance, hence increased chromatic distance would act to preserve the discrimination of given colours over a longer distance or possibly in turbid water. In the experiments conducted here, this effect is irrelevant due to very short distances between fish and target and the very clear water in aquaria.

We have demonstrated that the relationship between thresholds measured in the two directions in colour space agrees with the predictions of the RNL model [20,21], indicating the estimates of the relative value of receptor noise from anatomy can be used for modelling colour discrimination. An important consequence of our results is that the RNL model can be used for modelling colour discrimination of reef fish and applied to analysis of colourful patterns of reef fish in relation to detection and identification of fish by a fish [7].

## Data accessibility

Data and materials for this experiment are freely available in the PhD thesis ‘Color and spatial vision in a reef fish, *Rhinecanthus aculeatus*’ (see references section). Downloadable from the espace online repository at http://espace.library.uq.edu.au/view/UQ:282399.
